# Vasopressin-Induced Gangrene of the Bilateral Foot Digits and Right Index Finger Managed With Platelet-Rich Plasma Treatment

**DOI:** 10.7759/cureus.52229

**Published:** 2024-01-13

**Authors:** Aditya Pundkar, Sandeep Shrivastav, Rohan Chandanwale, Ankit M Jaiswal, Saksham Goyal

**Affiliations:** 1 Orthopedics, Jawaharlal Nehru Medical College, Datta Meghe Institute of Higher Education and Research, Wardha, IND

**Keywords:** infiltration, bilateral foot digit, angiogenesis, regeneration, prp

## Abstract

Bilateral foot digit gangrene generated by vasopressin is a serious complication for which management and treatment choices are extremely difficult. This case report presents a case of vasopressin-induced gangrene that was successfully treated with platelet-rich plasma (PRP) infiltration. A 20-year-old female patient came with a history of vasopressin treatment, causing bilateral foot digit gangrene and increasing necrosis. The patient's health quickly declined, and conventional care techniques had no effect on enhancing tissue perfusion or stopping the gangrene from getting worse. In our study, we have chosen to use PRP infiltration as an experimental therapeutic technique in light of the restricted choices available. This case study demonstrates the possibility of PRP infiltration as a cutting-edge and effective treatment for vasopressin-induced bilateral foot digit gangrene. The potential of PRP to stimulate angiogenesis, tissue regeneration, and wound healing is essential for optimizing the patient's results. For vasopressin-induced gangrene, more studies are required to evaluate the efficacy of PRP infiltration as a common therapy approach. This case study highlights the important role that PRP infiltration plays in enhancing tissue perfusion, stopping the advancement of necrosis, and promoting recovery.

## Introduction

An uncommon and serious complication known as vasopressin-induced dry gangrene is characterized by a reduced blood supply to the extremities, which can result in tissue necrosis and even the loss of limbs. Antidiuretic hormone, or vasopressin, is a hormone that regulates water balance and, when produced in excess, can cause blood vessels to constrict. Dry gangrene, characterized by a lack of infection and tissue liquefaction, is caused by impaired blood flow. The word "gangrene" refers to dead or dying bodily tissue that results from a local blood supply that is either insufficient or severe enough to kill the tissue. Gangrene has long been understood to represent a limited region of tissue death. The medical definition of gangrene encompasses any cause of tissue death resulting from an interruption in the blood supply. Wet gangrene is a type of gangrene most commonly associated with bacterial infection [1-3].

Debridement and intravenous antibiotics are commonly used in the early phases of gangrene therapy, and both methods are generally successful in treating the illness. If treatment is not received, gangrene can worsen and become life-threatening. Specifically, gas gangrene spreads quickly and has a high fatality rate from bloodstream infection [4]. Gangrene has become a global health concern, impacting morbidity and mortality rates across various ethnic groups. According to a study, the prevalence of gangrene in India ranges from 0% to 5% of the population, with the majority of affected individuals between the ages of 20 and 40 [5].

Platelet-rich plasma (PRP) has been suggested as a possible treatment for dry gangrene. PRP is isolated from the patient's whole blood, and the platelets are concentrated. Activated platelets found in PRP release a variety of growth factors and cytokines, such as platelet-derived growth factor, transforming growth factor-β (TGF-β), vascular endothelial growth factor (VEGF), basic fibroblast growth factor, insulin-like growth factor-1, and others. These compounds encourage tissue regeneration and repair [6]. It has been widely employed in several clinical and surgical procedures, showing encouraging outcomes in both experimental and clinical settings, especially in the healing of chronic wounds [7-9]. The crucial role platelets play in wound healing and tissue regeneration has been demonstrated by several studies. According to several research findings, PRP has a significant impact on vascularization. The vascularization of deep partial-thickness burns can be aided by PRP's increased VEGF release, improving the prognosis of burn wounds [10].

PRP does this by stimulating TGF-β1 expression, angiogenesis, and cell proliferation [11]. Moreover, PRP has been shown to accelerate the wound's local revascularization and stimulate the development of new capillaries in skin flap transplants [12,13]. Moreover, research has suggested that PRP can produce antibacterial agents, which would lessen local inflammation and stop wound infections [14].

## Case presentation

**Figure 1:**
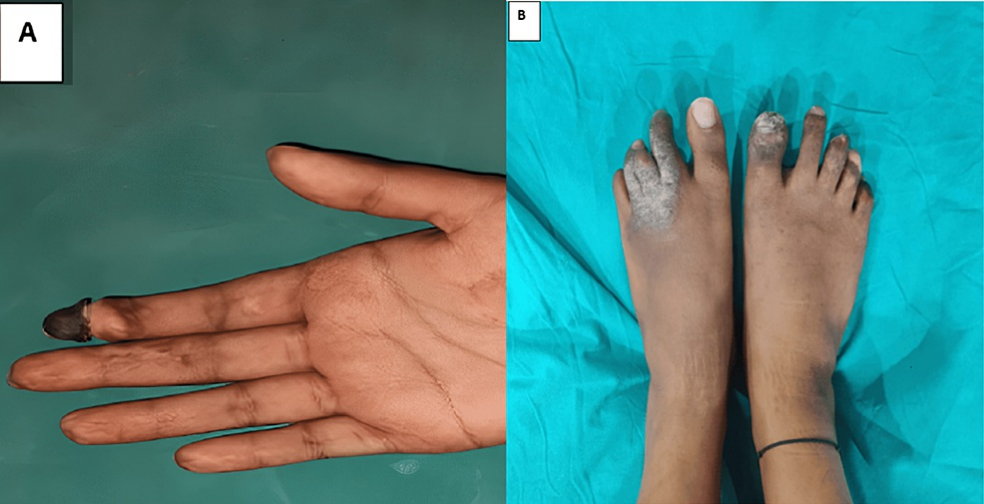
Gangrene of the digits of the (A) hand and (B) feet

A 20-year-old, non-diabetic, non-hypertensive female presented to the emergency department with complaints of fever with chills for two days, loose stools about 10-15 times per day, and multiple episodes of vomiting on and off for one day. Her heart rate was 110 beats per minute on general examination, and her blood pressure was 70/40 mmHg. She appeared pale and anicteric, with a dry tongue and cold extremities. She was managed with fluid resuscitation. Despite fluid resuscitation requiring large doses of inotropes such as vasopressin and noradrenaline, the patient remained hypotensive. She then had discomfort in both feet and the distal portion of her right index finger. The right index finger, toes, and fingers of the foot were blackened (Figure 1).

**Figure 2:**
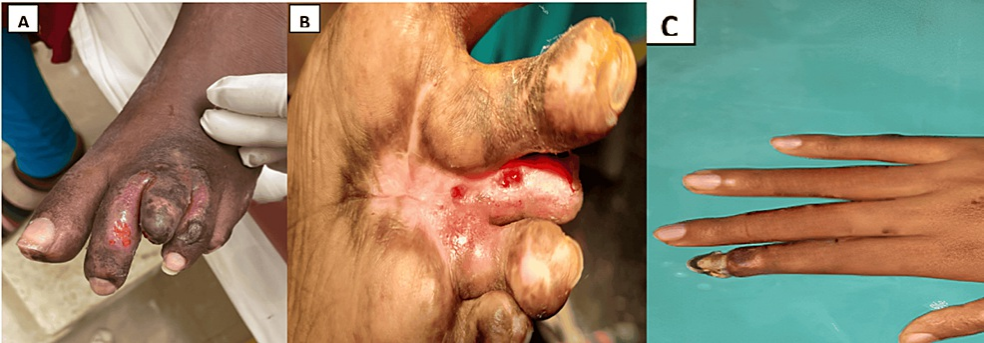
The condition of the digits after the initiation of platelet-rich plasma (PRP) treatment (A, B, and C)

The right index finger and toes had notable ischemia alterations in the patient. Concerning bilateral necrotic alterations to the right index fingers and toes, a dark purple discoloration was seen. Examining the lower limbs revealed diminished sensational feeling in the distal toes, but the dorsalis pedis and posterior tibial pulses were palpable. Upon performing upper extremity Doppler ultrasonography (US), bilateral normal artery flow was seen, with no indication of thrombosis. On the Doppler US scan of the lower limbs, normal velocities, bilateral biphasic flow, and the absence of a thrombus were observed. The ankle-brachial index for the lower limbs was within normal ranges. On hand and foot wound care, a consultation was held. It was decided to start the patient on PRP treatment. PRP injections were administered to the blackened areas of the hand and feet near the edges every fourth day. Dressing of the wound was done every second day with betadine and normal saline, and the wound was kept dry. Post initiation of treatment, blackening started reducing, as seen in Figure 2.

**Figure 3:**
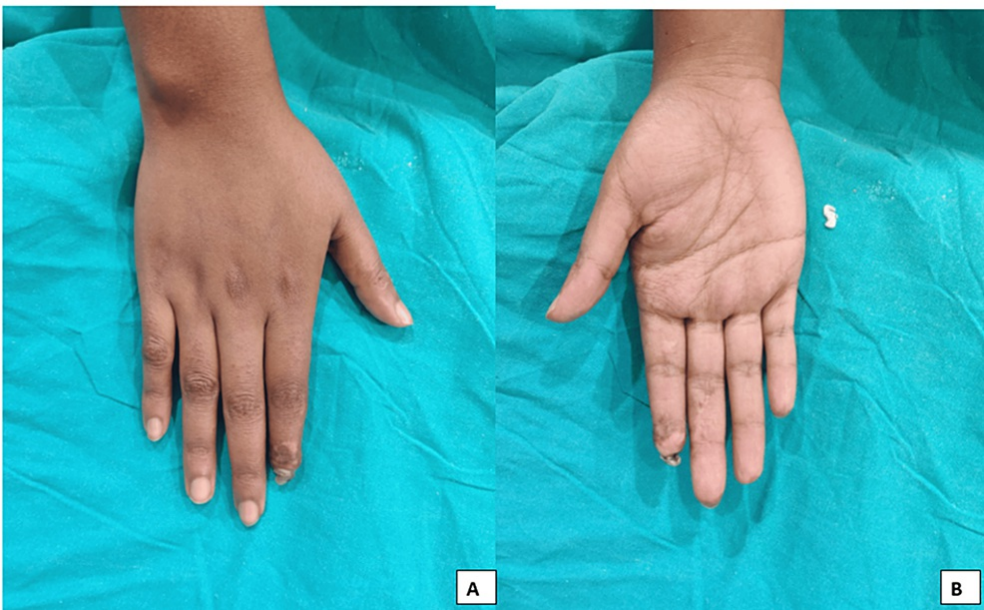
The condition of the index finger after completion of 10 platelet-rich plasma (PRP) cycles (A and B)

**Figure 4:**
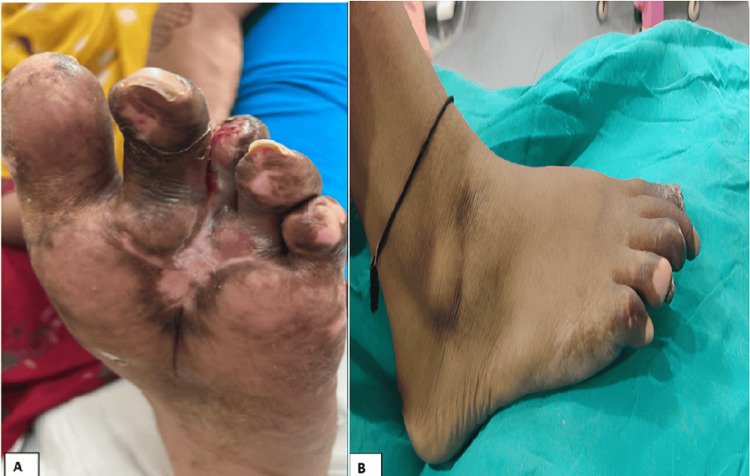
The condition of the foot digits after the completion of 10 platelet-rich plasma (PRP) cycles

A significant reduction of the blackening was seen, and there was a formation of granulation tissue after 10 cycles of PRP, as seen in Figures 3-4.

## Discussion

Since it has been linked to favorable results, norepinephrine remains the main therapy for septic shock. It is crucial to remember that end-organ hypoperfusion is a danger associated with norepinephrine [15]. Eight of the 153 research articles in the systematic review reported utilizing vasopressors in high dosages, defined as dosages greater than 0.5 mcg/kg/min. The treatment durations ranged from two to 84 hours, and the range of dosages employed was 0.58 to 4 mcg/kg/min [16].

Merely three of the eight cited investigations included necrosis or ischemia of the limbs and fingers [17]. Bilateral peripheral extremity gangrene without a significant arterial blockage is the hallmark of symmetrical peripheral gangrene (SPG). One known side effect of vasopressor usage in critically ill patients is distal extremity necrosis. Eighty of the 98 ICU patients in a retrospective analysis who were given vasoactive medications did so for phenylephrine (55%), norepinephrine (47%), ephedrine (31%), epinephrine (26%), and vasopressin (24%), which were the most prevalent [18]. Seventy of these patients had finger photoplethysmography; 40 had aberrant findings on one finger and 30 on the other. Five patients eventually needed amputations [19]. Consequently, it is recommended to routinely evaluate the skin for signs of SPG, including pallor, coldness, mottling, cyanosis, and pain in the distal extremities, when utilizing vasopressors in a clinical setting.

PRP has emerged as a potential treatment for various conditions, including tissue regeneration. PRP is a concentrated solution of platelets derived from the patient's own blood, containing a higher concentration of growth factors and bioactive substances that can support tissue healing and regeneration.

In theory, PRP might help treat dry gangrene by encouraging angiogenesis, the growth of new blood vessels, and tissue regeneration. PRP contains growth factors that can promote blood vessel creation, collagen production, and cell proliferation, all essential processes for tissue healing and repair. Apart from PRP, the management of dry gangrene often includes reversing or lowering vasopressin administration, enhancing blood flow, and controlling any related infections. It is important to emphasize that more investigation and clinical trials are needed to determine the precise procedure and efficacy of PRP in treating vasopressin-induced dry gangrene.

Neovascularization is essential for wound healing because it provides nutrients and oxygen. VEGF, a growth factor that binds to heparin, increases the proliferation of endothelial cells and attaches to specific receptors on the vascular endothelium to induce angiogenesis [20,21]. Studies have indicated that PRP promotes the production of VEGF and the proliferation of endothelial cells to improve angiogenesis in burns and trauma. Studies demonstrating that PRP promotes neovascularization and VEGF release in wounded tissue provide more proof of its beneficial effects on angiogenesis [22].

## Conclusions

This case report highlights the rare but severe complication of vasopressin-induced gangrene affecting the bilateral foot digits and right index finger. The timely recognition of this condition, prompt discontinuation of vasopressin, and the initiation of a comprehensive treatment approach played a pivotal role in the successful management of the patient. The use of PRP therapy demonstrated promising outcomes in promoting tissue regeneration and healing. This case emphasizes the importance of vigilance in monitoring for adverse effects associated with vasopressin use, especially in critical care settings, and suggests that PRP treatment could be a valuable adjunct in the management of vasopressin-induced gangrene. Further research and exploration of PRP's potential in similar cases could contribute to enhancing our understanding and refining treatment strategies for this uncommon yet challenging condition.
